# Clinical Evaluation of Humira^®^ Biosimilar ONS-3010 in Healthy Volunteers: Focus on Pharmacokinetics and Pharmacodynamics

**DOI:** 10.3389/fimmu.2016.00508

**Published:** 2016-11-28

**Authors:** Marlous R. Dillingh, Joannes A. A. Reijers, Karen E. Malone, Jacobus Burggraaf, Kenneth Bahrt, Liz Yamashita, Claudia Rehrig, Matthijs Moerland

**Affiliations:** ^1^Centre for Human Drug Research, Leiden, Netherlands; ^2^Good Biomarker Sciences, Leiden, Netherlands; ^3^Oncobiologics Inc., Cranbury, NJ, USA

**Keywords:** tumor necrosis factor-alpha, lipopolysaccharide, pharmacodynamics, pharmacokinetics, bioequivalence

## Abstract

ONS-3010 is being developed by Oncobiologics Inc. (Cranbury, NJ, USA) as a biosimilar of Humira^®^. This randomized, double blind, single-center phase I study (EudraCT registration # 2013-003551-38) was performed to demonstrate pharmacokinetic (PK) biosimilarity between two reference products (Humira^®^ EU and US) and ONS-3010 in healthy volunteers, and to compare the safety and immunogenicity profiles. In addition, the intended pharmacological activity was assessed and compared by application of a whole blood challenge. Hundred ninety-eight healthy volunteers received a single 40 mg subcutaneous dose of ONS-3010, Humira^®^ EU, or US. The pharmacodynamic effects were assessed by lipopolysaccharide (LPS)/aluminum hydroxide whole blood challenges (*n* = 36; *n* = 12 per treatment arm; male:female, 1:1). Equivalence was demonstrated on the PK endpoints (AUC_0–inf_, *C*_max_, and AUC_0–last_) based on bounds of 80–125% for the ratio of the geometric means (ONS-3010/Humira^®^). The immunogenicity profiles were comparable between treatment groups, and there were no indications for differences in routine safety parameters. Administration of adalimumab resulted in the observation of dramatically reduced tumor necrosis factor-α (TNFα) levels upon stimulation with LPS/aluminum hydroxide (>99%), with no differences between the three treatment groups in terms of magnitude or duration. Adalimumab also resulted in a reduction of LPS/aluminum hydroxide-induced interleukin (IL)-8 release (maximally 30%), suggested to have a causal relationship with the anti-TNFα treatment. LPS/aluminum hydroxide-induced release of IL-1β and IL-6 was not inhibited by anti-TNFα treatment. Taken together, these data are promising for the further clinical development of ONS-3010, demonstrate the relevance of the LPS/aluminum challenge to monitor Humira^®^ effects, and emphasize the value of whole blood challenges for monitoring of proximal drug effects in healthy volunteers, and potentially in the target population.

## Introduction

Biotherapeutic adalimumab (Humira^®^, AbbVie Inc., North Chicago, IL, USA) is a recombinant human IgG1 monoclonal antibody binding tumor necrosis factor-α (TNFα), which is one of the earliest and most potent cytokines mediating inflammatory responses (1). TNFα is produced primarily by activated macrophages but also by CD4+ lymphocytes, natural killer (NK) cells, neutrophils, mast cells, eosinophils, and neurons. Binding of TNFα to its receptor, TNF receptor type 1 (TNFR1) or TNF receptor type 2 (TNFR2), results in downstream activation of caspase, nuclear factor κ-B (NFκB), JNK, or MAPK pathways (2). TNFα is an autocrine stimulator as well as a potent paracrine inducer of interleukin (IL)-1, IL-6, IL-8, and other inflammatory cytokines (3). Blockage of TNFα therefore not only results in inhibition of direct TNF effects but also has a more general effect on inflammation (3, 4). Inactivation of TNFα has proven to be important in downregulating the inflammatory and immune reactions associated with rheumatoid arthritis and other autoimmune conditions (3, 5–10). Adalimumab binds specifically to TNFα, blocks its interaction with TNFR1 and TNFR2, and lyses surface TNFα-expressing cells *in vitro* in the presence of complement. Adalimumab also modulates biological responses induced or regulated by TNFα, including changes in the levels of adhesion molecules responsible for leukocyte migration (11).

The development of products that are designed as a biosimilar of the original licensed product has gained interest over recent years because of expiring patents of originator’s biotherapeutic products. This is also the case for Humira^®^: the patent is expected to expire in December 2016 in the US and in April 2018 in the EU. Per FDA and EMA guidelines, demonstration of biosimilarity between test and reference biotherapeutics comprises quality characteristics, pharmacokinetic (PK) properties, biological activity, safety, and efficacy (12–16). Although the FDA and EMA recommend to include pharmacodynamic (PD) endpoints in biosimilarity studies whenever feasible, no guidance is provided on the clinical development phase in which PD endpoints may be included. Assessing PD effects only in advanced phases of clinical development bears the risk of late discovery of non-biosimilarity, as was the case for Alpheon, recombinant human IFNα-2a (17). The addition of PD endpoints in early biosimilarity studies could add valuable information for the evaluation of overall comparability of products.

ONS-3010 is being developed by Oncobiologics Inc. (Cranbury, NJ, USA) as a biosimilar of Humira^®^. A phase I clinical study was performed to demonstrate PK biosimilarity between two reference products (the EU and US approved forms of Humira^®^) and ONS-3010 in healthy volunteers, and to compare the safety and immunogenicity profile of ONS-3010 with the two registered forms of Humira^®^. In addition, the intended pharmacological activity of the three products was assessed and compared by application of a whole blood challenge. In this model, robust activation of toll-like receptor (TLR)4-driven NFkB signaling and the NALP3/NLRP3 inflammasome pathway is induced in circulating immune cells by incubation of freshly isolated whole blood with lipopolysaccharide (LPS) and aluminum hydroxide (18–22). NFkB signaling generates an inflammatory response characterized by TNFα, IL-6, IL-8, IL-1β, and IFNγ release (23), whereas inflammasome activation results in further enhancement of IL-1β responses and secretion of IL-18 and IL-33 (21, 24, 25). TLR4 and inflammasome pathways are implicated in the pathogenesis of autoimmune diseases such as rheumatoid arthritis (26–28), and TNFα blockade specifically alters these responses by its effect on NFkB, playing a key role in NLRP3 priming (26, 29). Application of LPS/aluminum hydroxide whole blood challenges in this healthy volunteer trial not only allowed early assessment of the intended PD activity of ONS-3010 in comparison with the two reference products but also provided mechanistic insight into the secondary effects of TNFα blockade.

## Materials and Methods

### Study Design

The bioequivalence study was a randomized, double blind, single-center phase I study with three treatment arms: ONS-3010 (Oncobiologics Inc., Cranbury, NJ, USA) and reference products Humira^®^ EU (AbbVie, Berkshire, UK) and Humira^®^ US (AbbVie, North Chicago, IL, USA, EudraCT registration # 2013-003551-38). The study was performed in a total number of 198 volunteers, who were deemed healthy and screened negative for (latent) tuberculosis, acute infectious disease, malignancy, and autoimmune disorders. All women of child bearing potential and all males had to practice effective contraception during the study and had to be willing and able to continue contraception for at least 5 months after dose administration of study treatment to be able to participate in the study. In addition, the subjects had to have a negative screening result for Hepatitis B/C and HIV, and smoking was prohibited during the complete study period. Sixty-six subjects per treatment arm received a single subcutaneous dose of 40 mg TNFα antibody according to a randomization schedule, which was stratified for gender. PK, immunogenicity, and safety analyses were conducted for all 198 study participants. The PD effects of these three TNFα blockers were assessed by LPS/aluminum hydroxide whole blood challenges, for which 36 subjects were randomly selected from the 3 treatment arms (6 male and 6 female subjects per arm). Before the LPS/aluminum hydroxide whole blood challenges were implemented as a PD bioassay in the clinical study, the effect of TNFα blockade on the LPS/aluminum hydroxide-induced inflammatory response was explored in an *in vitro* experiment (see below).

The phase I clinical trial was conducted in accordance with the Declaration of Helsinki and Guideline for Good Clinical Practice and was approved by the Independent Ethics Committee of the Foundation “Evaluation of Ethics in Biomedical Research,” Assen, The Netherlands.

### Pharmacokinetic Analysis

Plasma concentrations of adalimumab (ONS-3010, Humira^®^ EU, or Humira^®^ US) following a single 40 mg subcutaneous dose administration were assessed in blood samples collected in 4 mL SST™ Gel and Clot Activator tubes (Becton Dickinson), from day 1 (pre-dose) up to day 71 (post-dose). An electrochemiluminescence assay (Meso Scale Discovery, MSD) was developed and validated to measure the concentration of adalimumab in human serum. Data values below the limit of quantification (BLOQ, 250 ng/mL) were set to 125 ng/mL (50% of the limit of quantification, LOQ) for summarizing and graphical purposes. If more than one-third of all values were BLOQ for a specific time point/treatment combination, the data point was not included in the summary/graph. For PK analysis, BLOQ data points were set to zero when not embedded between two measurable data points (i.e., >BLOQ) and occurring prior to *T*_max_. BLOQ data points were excluded from PK analysis when not embedded between two measurable data points and occurring after *T*_max_, or when embedded between two measurable data points.

### LPS/Aluminum Hydroxide Whole Blood Challenge

Preceding the phase I clinical trial, the effect of *in vitro* TNFα blockade on LPS/aluminum hydroxide-induced cytokine release in whole blood samples was explored. Blood specimens were obtained from four healthy male volunteers in sodium heparin tubes (Becton Dickinson). Fresh whole blood was (pre-)incubated in 96-well plates with Humira^®^ EU at 37°C at 5% CO_2_. The Humira^®^ concentration ranged from 0.3125 to 10 μg/mL, which was based on the expected maximum concentrations of 4.7 ± 1.6 μg/mL, observed after a single administration of 40 mg (11). After 90 min, 2 ng/mL LPS (Sigma-Aldrich, St Louis, MO, USA) and 100 μg/mL Alhydrogel 85 (aluminum hydroxide, Brenntag, Frederikssund, Denmark) were added to the samples followed by an additional incubation period of 20 h. In the clinical study, LPS/aluminum hydroxide whole blood challenges were performed in a subset of study participants, pre-dose (day 1) and on day 5, day 15, day 29, and day 64. Conditions for the whole blood challenges were similar as for the *in vitro* experiment above, except for the fact that the incubations were performed in a larger volume (2 mL), and no pre-incubation with Humira^®^ EU was performed. Cytokine release was measured in the culture supernatants by an electrochemiluminescence assay (MSD, V-plex; TNFα, IL-1β, IL-6, IL-8, and IFNγ, intra- and inter-assay variation: <10%) or ELISA (R&D Systems; IL-18).

### Immunogenicity

Blood samples for analysis of adalimumab-associated antibodies against ONS-3010 and Humira^®^ were collected in 4 mL SST™ Gel and Clot Activator tubes (Becton Dickinson). A bridging ELISA assay was used to measure anti-adalimumab antibodies in serum samples by electrochemiluminescence. Positive immunogenicity samples were further investigated to confirm the specificity of binding. If a sample was confirmed positive for specific anti-adalimumab antibodies, the neutralizing capacity of these antibodies was investigated. Binding specificity was determined by competitive inhibition with 5 mg/mL of unlabeled ONS-3010; samples with an inhibition >20.7% were confirmed positive for anti-drug antibody. Assay sensitivity was 2.463 ng/mL and inter-run assay precision 5.143–11.354%.

### Routine Safety Assessments

Pre-dose (day 1) and on day 2 (hematology only), day 4, day 8, day 36, and day 72, blood and urine samples were collected for routine hematology, biochemistry, and urinalysis safety parameter assessments. In addition, general safety measures such as vital signs, electrocardiography, and symptoms were assessed throughout the study.

### Data Analysis

Demographic and PD data were summarized and presented by graphical and tabular presentations. For the inhibition of TNFα and IFNγ release after incubation with LPS/aluminum hydroxide and increasing adalimumab concentrations *in vitro*, a model was fitted to the data in R (v2.15.2, R Foundation for Statistical Computing, Vienna, Austria, 2012) and described by a maximal effect (E_max_) and half maximal effect concentrations (EC_50_).

A non-compartmental analysis was performed to describe the PK of the antibodies using SOFTWARE. The area under the curve (AUC) was computed from zero to the last measurement point (AUC_0–last_). If the terminal phase was sufficiently well characterized (at least three data points after *T*_max_ and a linear regression *r*^2^ ≥0.5), the terminal half-life (*t*_1/2_) and the AUC zero to infinity (AUC_0–inf_) were estimated. PK profiles of the investigational product (ONS-3010) and the reference products (Humira^®^ EU and Humira^®^ US) were compared using general linear model procedures in SAS^®^. An analysis of variance (ANOVA) was performed on the untransformed elimination rate constant (*K*_el_), apparent terminal elimination half-life (*t*_1/2 el_), and ln-transformed AUC_0–last_, AUC_0–inf_, and *C*_max_. The ANOVA model included treatment as a fixed effect. The ratio of means with the 90% geometric confidence interval (CI) was calculated for AUC_0–last_, AUC_0–inf_, and *C*_max_. Bioequivalence was evaluated in accordance with EMA and FDA guidelines on the investigation of bioequivalence (30, 31). The hypothesis of bioequivalence for the PK parameters AUC_0–last_, AUC_0–inf_, and *C*_max_ was supported with 90% CI for the relative geometric means ratio fully enclosed in the equivalence bounds of 80–125%.

## Results

### Demographics

**Figure 1:**
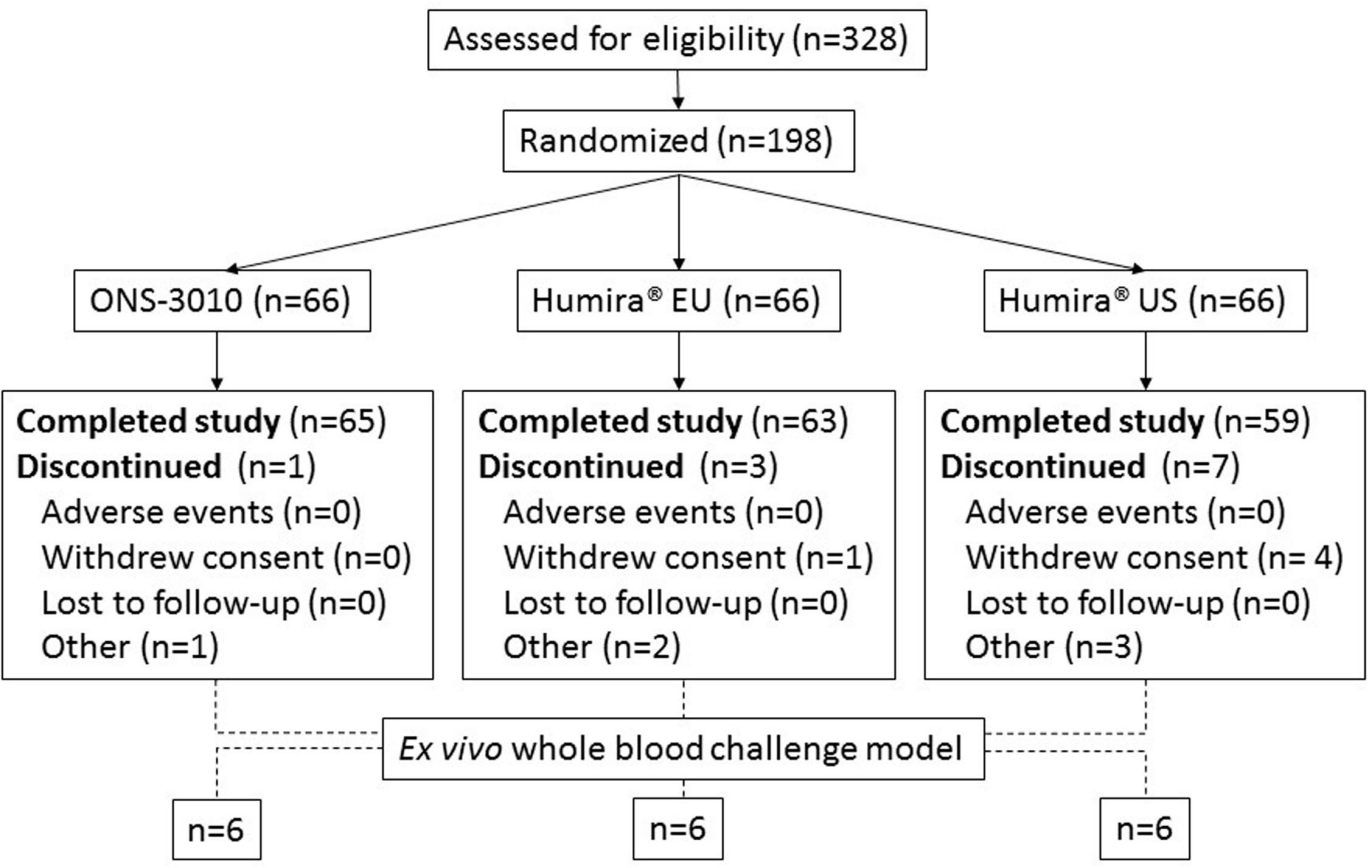
**Subject disposition**.

A total number of 90 male and 108 female subjects aged between 18 and 55 years, with a BMI of 19–29 kg/m^2^ and a body weight >50 kg were enrolled in the clinical trial with both sexes equally divided over the three treatment groups (ONS-3010 and Humira^®^ EU/US, Figure 1; Table 1).

**Table 1:** **Demographics and baseline characteristics**.

	Humira^®^ EU	Humira^®^ US	ONS-3010
**Age (years)**			
*N*	66	66	66
Mean (SD)	25.4 (8.4)	25.6 (9.0)	25.9 (9.3)
**Height (cm)**			
*N*	66	65	66
Mean (SD)	175.8 (8.6)	176.8 (9.5)	177.3 (8.4)
**Weight (kg)**			
*N*	66	66	66
Mean (SD)	72.0 (11.1)	72.4 (11.5)	71.5 (9.4)
**BMI (kg/m^2^)**			
*N*	66	66	66
Mean (SD)	23.2 (2.6)	23.1 (2.6)	22.7 (2.2)
**Sex**			
Female	36 (55%)	36 (55%)	36 (55%)
Male	30 (45%)	30 (45%)	30 (45%)
**Race**			
Asian	2 (3%)	0 (0%)	1 (2%)
Black or African American	7 (11%)	1 (2%)	5 (8%)
Hispanic or Latino	0 (0%)	2 (3%)	0 (0%)
White	50 (76%)	56 (85%)	58 (88%)
Mixed	6 (9%)	7 (11%)	2 (3%)
Other	1 (2%)	0 (0%)	0 (0%)

### Pharmacokinetic Analysis

Five subjects (four Humira^®^ US; one Humira^®^ EU) had an incomplete PK profile as a result of early study termination, and therefore PK parameters could not be determined accurately. These participants were excluded from the statistical analysis on total exposure parameters (e.g., AUC_0–last_). Two of these subjects had a PK profile up to 7 and 8 days, based on which *C*_max_ and *T*_max_ could be determined, and these data were included in the statistical analysis (Table 2).

**Table 2:** **Non-compartmental pharmacokinetic analysis**.

	ONS-3010 (*n* = 66)	Humira^®^ EU (*n* = 66)	Humira^®^ US (*n* = 66)
AUC_0–inf_ (ng × h/mL)	2,680,234 (995,883)	2,631,077 (1,077,199)	2,577,967 (1,112,632)
AUC_0–last_ (ng × h/mL)	2,325,364 (870,647)	2,376,523 (891,150)	2,304,145 (941,728)
*C*_max_ (ng/mL)	3869 (842)	3898 (987)	3692 (1030)
*T*_max_ (h)	146 (59)	134 (70)	152 (82)
*t*_1/2_ (h)	317 (191)	307 (178)	318 (194)

*Mean (SD)*.

**Figure 2:**
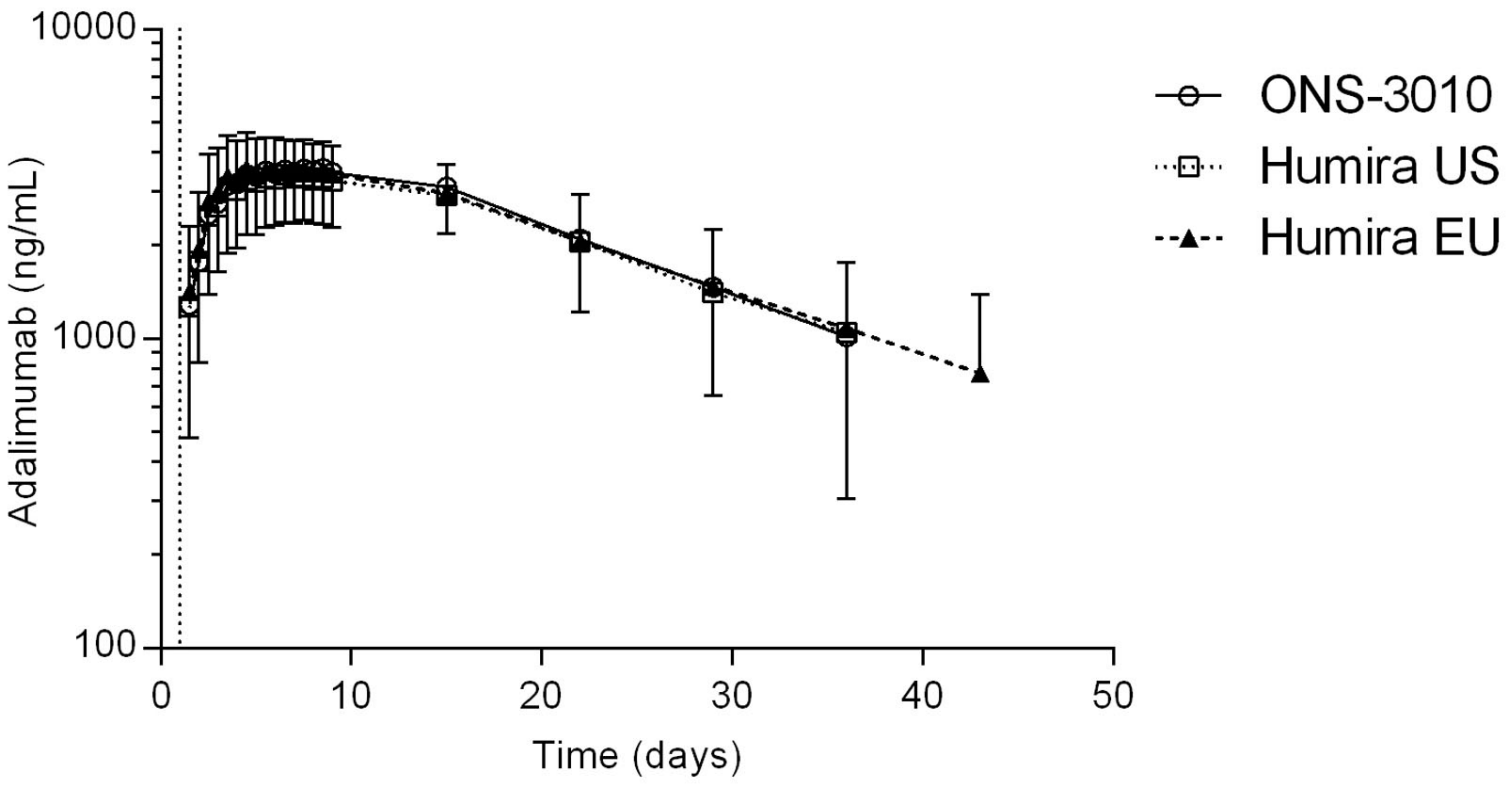
**Adalimumab concentration-time profile for ONS-3010, Humira^®^ EU, and Humira^®^ US with SD; vertical line at day 1 denotes dosing**.

Subcutaneous administration of adalimumab resulted in a steady increase in plasma concentrations starting at approximately 36 h post-dose to reach a maximum concentration after 6–7 days, followed by a log-linear decrease and a non-linear decrease once the plasma concentration reached lower levels, similar to other monoclonal antibodies (Figure 2). Large variability in antibody concentration was observed between subjects during the elimination phase, with some subjects having adalimumab plasma concentration levels BLOQ (<250 ng/mL) 5 weeks after dosing, while others continued to have measurable levels up to the last study visit (day 71). On the primary PK endpoints, AUC_0–inf_ and *C*_max_, equivalence was demonstrated based on bounds of 80–125% for the ratio of the geometric means (ONS-3010/Humira^®^; Table 3). Equivalence was also demonstrated for secondary PK endpoint AUC_0–last_.

**Table 3:** **Non-compartmental pharmacokinetic analysis – contrasts of ANOVA**.

	Humira^®^ EU vs. Humira^®^ US			ONS-3010 vs. Humira^®^ US			ONS-3010 vs. Humira^®^ EU		
	Contrast	90% CI	*p*-value	Contrast	90% CI	*p*-value	Contrast	90% CI	*p*-value
AUC_0–inf_ (ng × h/mL)	1.04	0.92–1.17	0.6365	1.06	0.94–1.20	0.4061	1.03	0.91–1.16	0.7141
AUC_0–last_ (ng × h/mL)	1.05	0.92–1.20	0.5118	1.01	0.89–1.15	0.8705	0.96	0.85–1.09	0.6160
*C*_max_ (ng/mL)	1.07	0.99–1.15	0.1811	1.06	0.98–1.15	0.1899	1.00	0.92–1.08	0.9746

### LPS/Aluminum Hydroxide Whole Blood Challenge

**Figure 3:**
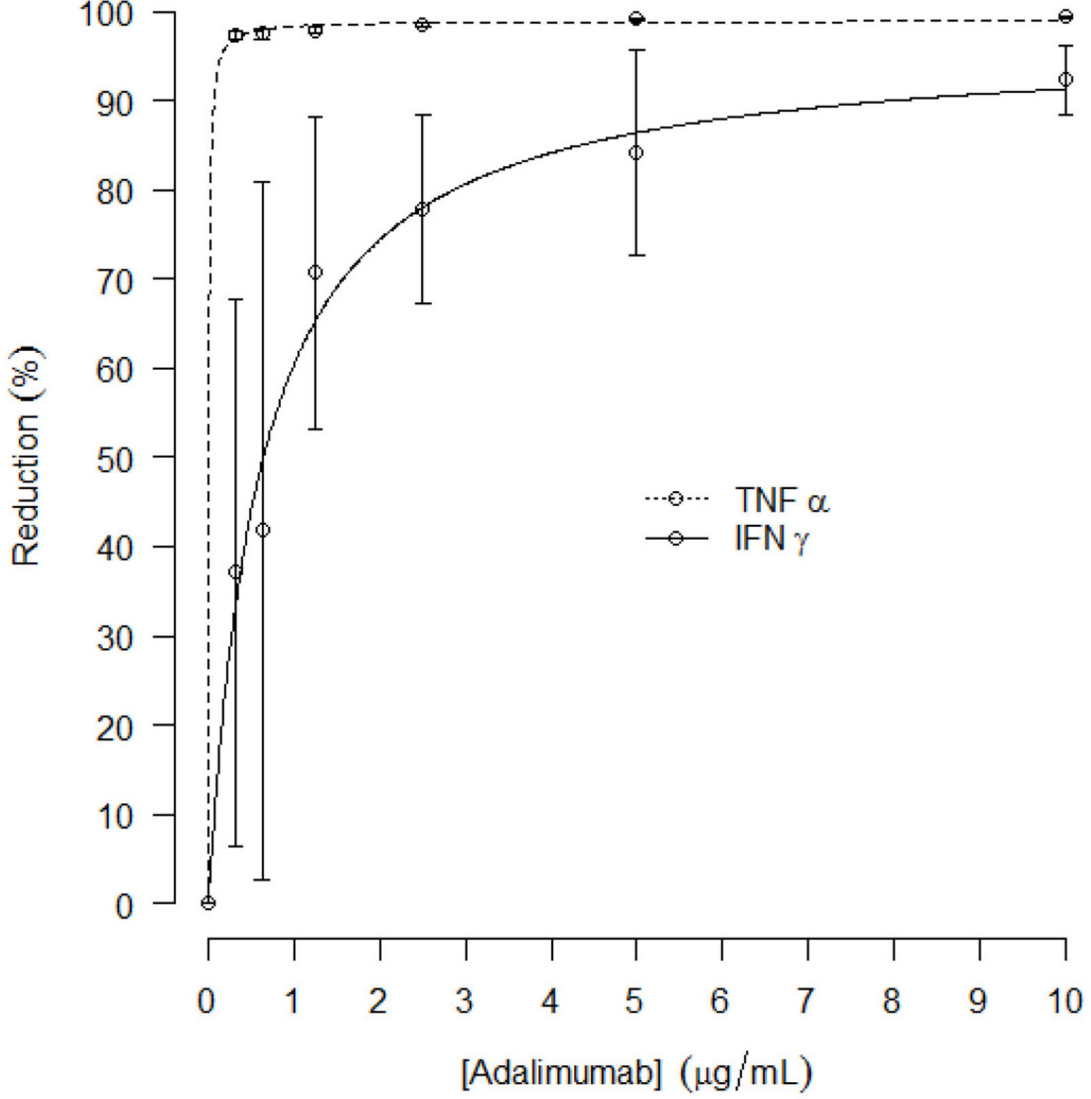
**Mean reduction of *in vitro* TNFα and IFNγ release (%, with SD) after LPS/aluminum hydroxide stimulation and incubation with increasing concentrations of adalimumab; curve fit presented as dotted line**.

In a separate experiment preceding the clinical study, the effect of *in vitro* TNFα blockade on LPS/aluminum hydroxide-induced cytokine release in whole blood samples was explored. Addition of adalimumab to the LPS/aluminum hydroxide-triggered whole blood samples resulted in dramatically reduced TNFα levels measured in the culture supernatant. An average reduction in TNFα level of 97 ± 0.6% was observed already at the lowest adalimumab concentration evaluated (0.3125 μg/mL), as compared to the LPS/aluminum hydroxide only samples. With increasing concentrations of adalimumab, further reductions in TNFα levels were measured, up to 99 ± 0.1%. In addition, TNFα blockade affected the LPS/aluminum hydroxide-induced release of IFNγ with a maximal reduction of 93 ± 4% observed at an adalimumab concentration of 10 μg/mL. The adalimumab concentration-effect curves for TNFα and IFNγ are presented in Figure 3, with an E_max_ of 99 and 97% and an EC_50_ of 0.006 and 0.6 μg/mL for TNFα and IFNγ, respectively. Increasing concentrations of adalimumab did not affect the release of IL-6, IL-1β, and IL-18 following LPS/aluminum hydroxide stimulation of whole blood (data not shown).

**Figure 4:**
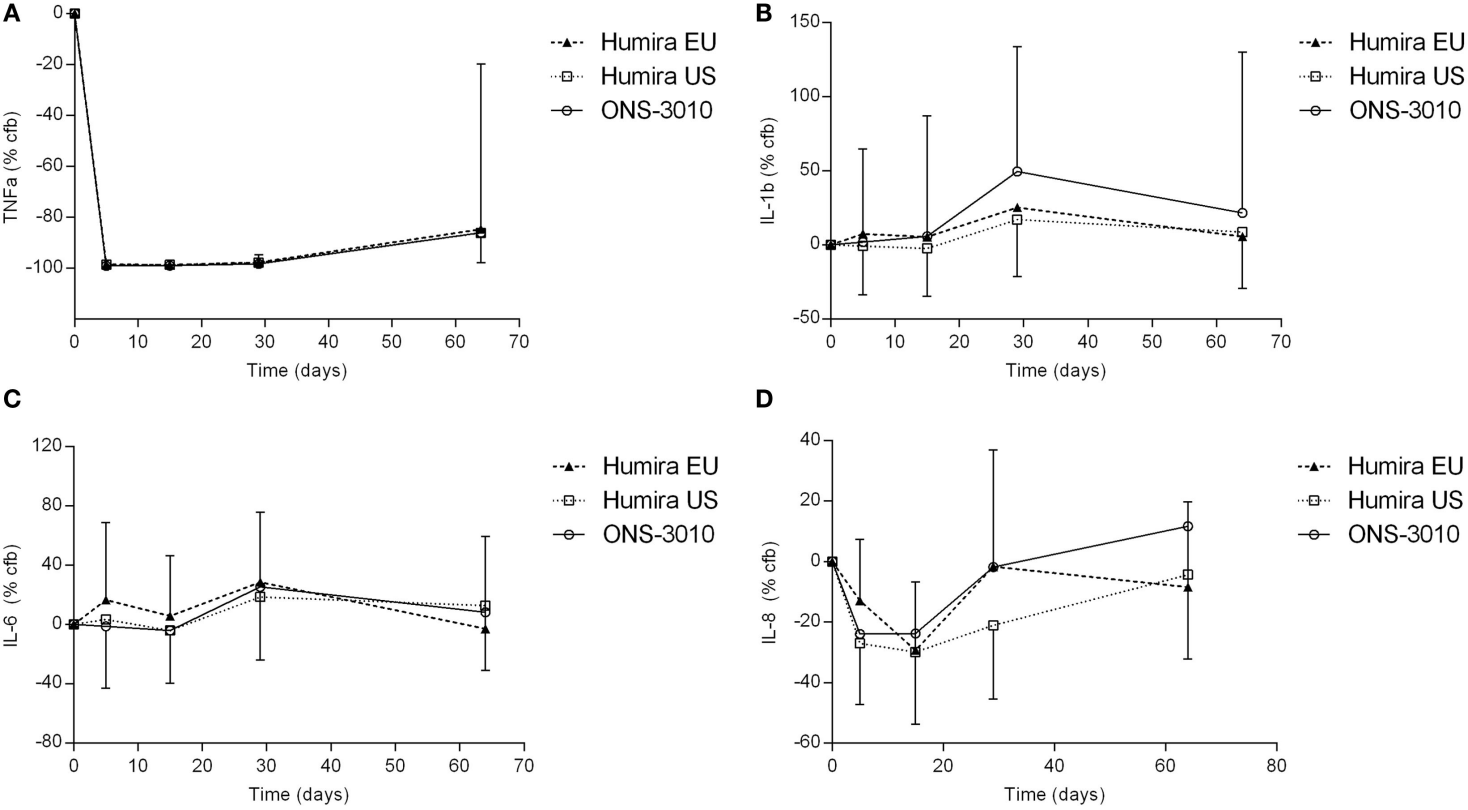
**TNFα (A), IL-1β (B), IL-6 (C), and IL-8 (D) release after *ex vivo* LPS/aluminum hydroxide stimulation of blood samples collected from clinical study participants [change from baseline (cfb) %, with SD]**.

In the clinical study, adalimumab treatment resulted in a more than 99% reduction in measurable TNFα levels in LPS/aluminum hydroxide-triggered whole blood samples (Figure 4A). This effect was maximal at the first time point investigated (day 5) and lasted until at least 3 weeks post-dose. Thereafter, TNFα levels started to return to baseline with individual time courses depending on the adalimumab plasma concentration. On average, TNFα levels were still approximately 85% reduced at the last time point investigated (day 64). The reduction in TNFα levels did not differ between the three treatment groups.

Lipopolysaccharide/aluminum hydroxide-induced release of IL-1β and IL-6 was not inhibited by TNFα blockade (Figures 4B,C). For all treatment groups, a slight increase over baseline cytokine levels was observed on day 29, both for IL-1β (50, 25, and 17% for treatment ONS-3010, Humira^®^ EU, and Humira^®^ US, respectively) and IL-6 (25, 29, and 19%). Administration of adalimumab resulted in a reduction of LPS/aluminum hydroxide-induced IL-8 release (Figure 4D). Minor differences were observed between treatment groups, with maximal reductions observed on day 5 [ONS-3010, −24% (CV 56%)] and day 15 [Humira^®^ EU, −29% (CV 28%); Humira^®^ US, −30% (CV 44%)], and a return to baseline levels between day 29 and day 64. Whereas adalimumab inhibited LPS/aluminum hydroxide-induced IFNγ release in the preclinical *in vitro* experiment, this effect could not be confirmed in the clinical study. IFNγ release was highly variable between subjects and BLOQ (<114 pg/mL) in the baseline samples of 21 out of the total number of 36 participants. IFNγ release strongly differed between males and females with mean baseline values of 1308 ± 1803 and 144 ± 278 pg/mL, respectively. Also, for the other cytokines (TNFα, IL-1β, IL-6, and IL-8) sex differences were observed, but to a lesser extent (cytokine release on average 1.6-fold higher in the male subjects, data not shown).

### Immunogenicity and Immune Cell Counts

The immunogenicity profiles were well comparable between the ONS-3010, Humira^®^ EU, and Humira^®^ US treatment groups for the confirmed and neutralizing antibodies (Table 4).

**Table 4:** **Confirmed and neutralizing antibodies**.

	Confirmed antibodies			Neutralizing antibodies		
	ONS-3010 (*n* = 63)^a^	Humira^®^ EU (*n* = 62)^a^	Humira^®^ US (*n* = 65)^a^	ONS-3010 (*n* = 66)	Humira^®^ EU (*n* = 65)^b^	Humira^®^ US (*n* = 66)
Day 15	20	17	13	8	8	3
Day 43	31	28	25	31	24	24
Day 57	39	38	37	41	37	37

*Samples with normalized inhibition <0.886 were deemed positive for neutralizing antibodies. Subjects with a positive result for confirmed (^a^) and neutralizing (^b^) antibodies pre-dose were excluded*.

**Figure 5:**
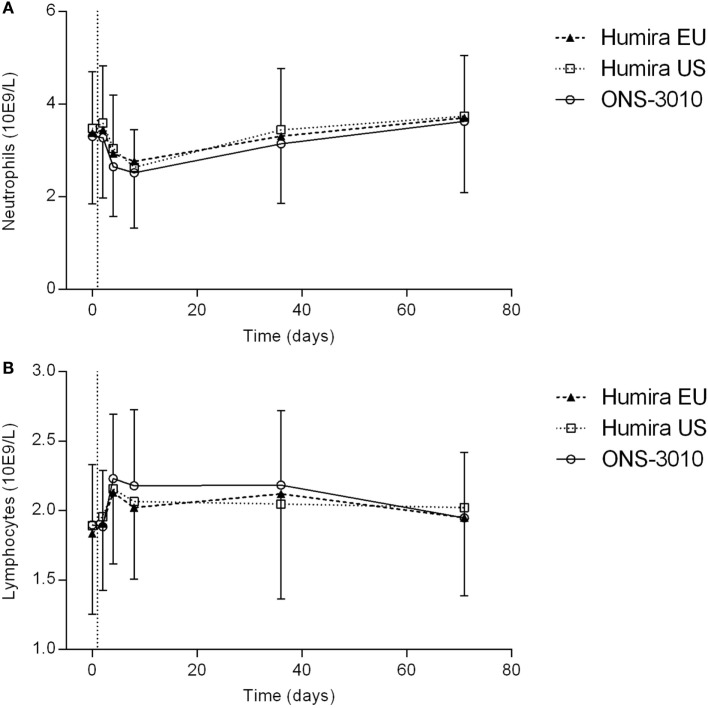
**Neutrophil (A) and lymphocyte (B) count with SD; vertical line at day 1 denotes dosing**.

Adalimumab administration resulted in a mild decrease in neutrophil count, amounting maximally 15–20% at day 8, and returning to baseline levels after 5 weeks (Figure 5A). Furthermore, adalimumab administration induced a mild increase in lymphocyte count, peaking 3 days after dose administration (Figure 5B). There were no indications for differences between treatment groups in neutrophil and lymphocyte cell counts over time.

## Discussion

A phase I clinical study was performed to demonstrate biosimilarity between ONS-3010 and two reference adalimumab products (the EU and US approved forms of Humira^®^) in healthy volunteers. For the PK endpoints (AUC_0–inf_, *C*_max_, AUC_0–last_) equivalence was demonstrated between ONS-3010 and the marketed products, based on bounds of 80–125% for the ratio of the geometric means (ONS-3010/Humira^®^). The immunogenicity profiles were comparable between treatment groups for the confirmed and neutralizing antibodies, and there were no indications for differences between treatment groups in routine safety parameters, including neutrophil and lymphocyte cell counts. The adverse events (AEs), which were reported most frequently (probably, possibly, or unlikely related to treatment) were a burning sensation upon injection at injection site, headache, and nasopharyngitis. In general, the AEs were evenly divided over the treatments, mild in severity, and self-limiting. In addition to PK, safety, and immunogenicity, the intended pharmacological activity of the compounds was assessed in this study using whole blood LPS/aluminum challenges. This PD endpoint was included to reduce the risk of late discovery of bio-non-similarity for the intended drug effect, even though this approach is rarely applied in biosimilarity trials in healthy volunteers. An LPS/aluminum challenge was used to induce TLR4-driven NFkB signaling and NALP3/NLRP3 inflammasome activation. Both pathways are implicated in the pathogenesis of autoimmune diseases for which Humira^®^ is being prescribed.

Preceding the clinical study, the effect of TNFα blockade on LPS/aluminum hydroxide-induced cytokine release in whole blood samples was verified *in vitro*. TNFα blockade resulted in dramatically reduced TNFα levels (maximally 99%) measured in the culture supernatant. IFNγ release was also strongly affected, with a maximal reduction of 93%. Previous studies also described a reduced production of IFNγ following the use of TNFα blockers. However, the underlying mechanism for the observed inhibition is not fully elucidated (32–34). IFNγ can be produced by a variety of cells including T- and B-lymphocytes, antigen-presenting cells, and NK cells (35). NK cells are probably an important source of IFNγ in our model. IFNγ production is NFκB-driven and supported by IL-12 and IL-18 (36–38). TNFα may act synergistically with these cytokines, explaining the reduced IFNγ release observed in our experiment.

In contrast to the effect on IFNγ release, *in vitro* TNFα blockade did not modulate the release of IL-1β, IL-6, and IL-18. Although TNFα-induced NFκB signaling drives transcription of various cytokines, the unaffected IL-1β, IL-6, and IL-18 release upon TNFα blockade suggests that primary LPS-driven responses are sufficient to induce maximal cytokine release, and that secondary signaling *via* LPS-induced TNFα does not augment the LPS-driven response.

The adalimumab induced reduction in TNF levels upon stimulation with LPS/aluminum hydroxide could be reproduced in the clinical samples of ONS-3010 and Humira^®^ treated subjects. Treatment resulted in a more than 99% reduction in detectable TNFα levels in the whole blood culture supernatant. The reduction in TNFα levels did not differ between the three treatment groups in terms of magnitude or duration. Surprisingly, whereas adalimumab inhibited LPS/aluminum hydroxide-induced IFNγ release in the preclinical *in vitro* experiment, this effect could not be confirmed in the clinical study. As described in the Section “Results,” the inter-individual variability in IFNγ levels was substantial, and a large number of data points was BLOQ (<114 pg/mL). Strikingly, a major difference in IFNγ release was observed between male and female subjects (baseline levels 1308 and 144 pg/mL, respectively). These data are in contrast to studies reporting no significant differences in IFNγ cytokine levels between both sexes (39, 40), and we are not aware of studies reporting the existence of sex-dependent IFNγ release. The different responsiveness in IFNγ release observed in the current study upon TLR4/inflammasome stimulation may relate to sex differences in circulating cell populations producing IFNγ or hormonal influences that play a role in this specific immune response (41). IL-1β, IL-6, and IL-8 responses were comparable for the three treatment groups. In line with the results from the preclinical *in vitro* experiment, LPS/aluminum hydroxide-induced release of IL-1β and IL-6 was not inhibited by anti-TNFα treatment in the clinical study. In contrast, administration of adalimumab did result in a reduction of LPS/aluminum hydroxide-induced IL-8 release. A maximal decrease of approximately 30% was observed at day 15. Although this effect size is limited and the inter-individual variability relatively large, the timing of the IL-8 decrease suggests a causal relationship with the anti-TNFα treatment. This observation may be explained by the role of TNFα as an autocrine stimulator and a potent paracrine inducer of inflammatory cytokines (3), although the duration of the IL-8 reduction (i.e., shorter than the TNFα reduction) and the absent effect on IL-6 release raises questions about the responsible mechanism.

The advised Humira^®^ dose for the treatment of rheumatoid arthritis is 40 mg (11). Our data demonstrate that 40 mg adalimumab results in maximally reduced TNFα levels in LPS-/aluminum-exposed whole blood cultures, persisting for at least a month (Figure 4A). During this period, the corresponding adalimumab concentrations ranged from 3800 (*T*_max_) to 1050 ng/mL (day 36, Figure 2). A comparable adalimumab concentration range also resulted in a maximal reduction of TNFα levels in our preclinical *in vitro* experiment (Figure 3), which demonstrates the excellent translatability between *in vitro* and *ex vivo* TNFα blocking in an LPS/aluminum challenge model.

Importantly, our *in vitro* TNFα blockade experiment shows that a dose of 40 mg Humira^®^, which is applied in clinical practice, is a rational dose based on the observed PD effects in this study: circulating adalimumab concentrations below 1 μg/mL (Figure 2, after day 36) translated into a sub-maximal reduction of LPS-/aluminum-induced TNFα levels (Figure 4). Taken together, these data demonstrate the relevance of the LPS/aluminum challenge to monitor Humira^®^ effects and emphasize the value of whole blood challenges for monitoring of proximal drug effects in healthy volunteers, and potentially in the target population.

In conclusion, this healthy volunteer study demonstrated equivalence for PK endpoints and closely comparable PD biomarker profiles between ONS-3010 and the marketed Humira^®^. The immunogenicity profiles were well comparable between treatment groups, and there were no indications for differences in routine safety parameters. These data are promising for the further clinical development of ONS-3010 and underline the value of incorporation of PD measures in early clinical phase biosimilar trials.

## Ethics Statement

The study was approved by the Independent Ethics Committee of the Foundation “Evaluation of Ethics in Biomedical Research,” Assen, The Netherlands. Subjects were given oral and written information about the study prior to medical screening. The subjects were permitted to ask questions to qualified staff and were given ample opportunity to carefully consider participation in the trial. After they gave written acknowledgement of informed consent to participate, a medical screening took place.

## Author Contributions

Oncobiologics Inc. conceptualized the study. MM, JB, and LY designed the study. KM was involved in the design of the laboratory methods and carried out the laboratory experiments. MM and JB supervised the complete study. MD, JR, MM, and JB were responsible for the clinical execution of the study and interpretation of the results. All the authors (MD, JR, KM, JB, KB, LY, CR, and MM) were involved in writing the manuscript and approved the final manuscript.

## Conflict of Interest Statement

This study was funded by Oncobiologics, Inc. CR, KB, and LY are employees of Oncobiologics Inc. All authors received clinical trial support from Oncobiologics, Inc. MD, JR, JB, and MM are employees of the Centre for Human Drug Research, responsible for the study conduct. KM was employee of Good Biomarker Sciences and was responsible for the performance of the laboratory experiments. They received no personal remuneration.

## Abbreviations

ANOVA, analysis of variance; AUC, area under the curve; AUC_0–last_, AUC zero to last measurement point; AUC_0–inf_, AUC zero to infinity; BLOQ, below the limit of quantification; CI, confidence interval; *C*_max_, maximum concentration; EC_50_, half maximal effect concentration; ELISA, enzyme-linked immunosorbent assay; EMA, European Medicines Agency; E_max_, maximal effect; FDA, Food and Drug Administration; IFNγ, interferon-γ; IL-1β, interleukin-1β; IL-6, interleukin-6; IL-8, interleukin-8; IL-12, interleukin-12; IL-18, interleukin-18; *K*_el_, elimination rate constant; LPS, lipopolysaccharide; MSD, Meso Scale Discovery; NALP3, NACHT, LRR, and PYD domains-containing protein 3; NLRP3, NACHT, LRR, and PYD domains-containing protein 3 encoding gene; NFκB, nuclear factor κ-B; NK, natural killer; PD, pharmacodynamics; PK, pharmacokinetics; *t*_1/2_, terminal half-life; TLR4, toll-like receptor-4; *T*_max_, time at observed maximum concentration; TNFα, tumor necrosis factor-α; TNFR1, TNF receptor type 1; TNFR2, TNF receptor type 2.
